# Experimental Evaluation of Fecal‐Mediated Transfer of *Salmonella* Typhi From *Blattella germanica* to Mice

**DOI:** 10.1155/jotm/5010895

**Published:** 2026-05-31

**Authors:** Dong-fen Geng, Teng Zhao, Xiao-hui Liu, Rui-xiang Zhang, Hao-tian Yu, Xiao-li Chen, Dan Xing, Jia-hong Wu, Chun-xiao Li

**Affiliations:** ^1^ School of Basic Medical Sciences, Guizhou Medical University, Guiyang, China, gmc.edu.cn; ^2^ State Key Laboratory of Pathogen and Biosecurity, Beijing, China

**Keywords:** *Blattella germanica*, mammals, *Salmonella* enterica serovar Typhi, transmit

## Abstract

*Blattella germanica* (German cockroaches) are recognized as potential carriers of intestinal pathogens, yet their role in delivering human pathogens to mammals via fecal contamination has not been experimentally evaluated. In this study, we established an experimental model using 100 *B. germanica* infected with a fluorescently labeled *Salmonella enterica* serovar Typhi Ty21a strain and assessed their ability to contaminate food and water. Fluorescent signals were detected in both midgut tissues and fecal matter of cockroaches within 6 h postinfection, peaking at 1 day (midgut: 4.52 × 10^10^ relative fluorescence unit [RFU] and feces: 6.12 × 10^8^ RFU) and persisting up to 7 days. Importantly, cockroach‐derived fecal suspensions administered to BALB/c mice resulted in rapid and transient intestinal exposure, with detectable bacterial signals in mouse feces within 30 min (1.13 × 10^3^ to 1.08 × 10^4^ RFU) and in intestinal tissues for up to 12 h; no fluorescent signal was detected thereafter (up to 7 days). Fluorescence quantification showed a positive correlation with viable bacterial counts (CFUs) (*R* ^2^ = 0.78, *p* < 0.05), supporting use of RFU as a proxy for Ty21a sfgfp abundance. Collectively, these findings support the potential role of *B. germanica* as a mechanical vector that can contaminate food and water and facilitate fecal–oral exposure of mammals to *Salmonella* under enclosed conditions.

## 1. Introduction

In recent years, the spread of insect‐borne diseases has been expanding due to multiple factors, including climate change, urbanization, land reclamation, and population growth, as well as evolving lifestyles and transportation patterns [1, 2]. These diseases account for approximately 5%–10% of all infectious disease cases in China annually, yet they are responsible for nearly 30%–40% of infectious disease‐related deaths, posing a significant threat to public health security [3–6]. Among enteric pathogens, *Salmonella* remains a leading cause of foodborne illness and outbreaks worldwide and continues to pose challenges due to antimicrobial resistance and evolving epidemiology [7–9].

Cockroaches, having existed for over 300 million years, have showed remarkable environmental adaptability and reproductive capacity throughout their evolutionary history [10]. There are over 4300 known cockroach species in the world, of which approximately 30 species have developed close associations with human habitats [11]. In China, the most prevalent cockroach species include the *Blattella germanica* (German cockroach), *Periplaneta americana* (American cockroach), and *Periplaneta australasiae* (Australian cockroach) [12]. These species predominantly inhabit dark, warm, and humid environments, often in unsanitary conditions. As omnivorous scavengers, cockroaches exhibit remarkable dietary flexibility, feeding on diverse organic matter ranging from food waste to human and animal secretions and excretions [13–16]. Although *B. germanica* has been identified as a potential vector for various pathogenic microorganisms [17, 18], scientific understanding of its direct role in pathogen infection pathways remains limited [19, 20]. Notably, studies have revealed that approximately 20% of *B. germanica* populations carry *Salmonella*, highlighting their potential public health significance [21, 22]. Emerging research showed that *B. germanica* has the potential to transmit *Salmonella*, particularly in densely populated urban settings where infestations frequently coincide with compromised sanitation conditions [22–24]. These findings establish *B. germanica* as potential reservoirs for pathogenic microorganisms, particularly in environments where poor sanitation practices intersect with dense human populations. Synanthropic cockroaches commonly infest homes, food‐handling establishments, hospitals, and livestock facilities, creating interfaces for human–environment–animal pathogen exchange [25–28].

A study conducted by Graffar and Mertens at a pediatric hospital in Brussels, Belgium, showed a nosocomial outbreak of nontyphoidal salmonellosis, highlighting the limitations of conventional infection control measures—including patient isolation protocols and environmental disinfection procedures—in containing the pathogen infection [23, 29]. Furthermore, researchers in Australia have reported that *B. germanica* can carry *Salmonella* in hospital environments [30]. These findings show the capacity of *B. germanica* to act as vectors for *Salmonella* infection in urban environments. Additional research has showed that *Salmonella*‐infected specimens can horizontally transfer pathogenic strains to conspecifics within colonies, albeit with limited infection efficacy under laboratory conditions [17].

*Salmonella*‐infected *B. germanica* primarily transmits the pathogen through two distinct routes: mechanical transfer through external surface contamination and fecal–oral dissemination through bacterial excretion [31]. The results demonstrated that orally ingested *Salmonella* can persist in the cockroach gut for at least 7 days, with concurrent bacterial shedding observed in fecal matter through microbiological analysis [24, 31, 32]. The primary infection route within cockroach populations occurred through individuals contaminating communal food and water sources [31, 33, 34].

These studies suggest that *B. germanica* may contribute to *Salmonella* dissemination; however, the mechanistic links between cockroach infection, environmental contamination, and mammalian exposure remain incompletely defined. To address this gap, we established a controlled laboratory model using *B. germanica* colonies and a fluorescently tagged attenuated strain of *Salmonella enterica* serovar Typhi (Ty21a), enabling visualization‐based quantification of bacterial persistence and shedding. In addition, recent advances in *Salmonella* detection and molecular typing—such as molecular assays and whole‐genome sequencing—have strengthened source tracking and risk assessment, providing an important context for interpreting cockroach‐associated transmission pathways [35–38]. Our results provide experimental support for a plausible cockroach‐associated fecal–oral exposure pathway under enclosed conditions and inform vector control and sanitation strategies.

## 2. Materials and Methods

### 2.1. Cockroach

In this experiment, *B. germanica* from laboratory colonies were maintained under standardized environmental conditions: temperature at 26 ± 1°C, relative humidity of 50 ± 10%, and a 12:12 h light–dark (L:D) photoperiod. The rearing environment was supplemented with free access to tap water, rodent chow, and vertically oriented wooden shelters to simulate natural habitat. Sex‐specific segregation was implemented, with adult males (*n* = 100) selected for experimental procedures while maintaining a separate cohort of gravid females for colony propagation.

### 2.2. Construction and Culture Conditions of *Salmonella* Typhi Ty21a sfgfp Strain

The Ty21a strain utilized in this study was commercially sourced from Shanghai SIG Biotechnology Co., Ltd. and labeled with green fluorescence by Hangzhou Baosai Biotechnology Co. A genetically engineered variant expressing green fluorescent protein (GFP) was developed through CRISPR‐Cas9‐mediated genome editing. The GFP coding sequence (sfgfp) was integrated downstream of the conserved glms locus in Ty21a, facilitated by homologous recombination. Specifically, homology arms were synthesized using Ty21a genomic DNA as template, followed by construction of the glms‐sfgfp‐PUC19 plasmid through PCR amplification and seamless cloning. The sfgfp cassette was subsequently amplified from this plasmid and inserted into the homology arm region.

The glms‐sfgfp homology arm and glms‐specific sgRNA plasmid were coelectroporated into Cas9‐expressing Ty21a competent cells. Transformants were selected on kanamycin plates, with successful sfgfp knock‐in confirmed through sequencing analysis. Plasmid clearance was achieved through IPTG induction, followed by thermal elimination of the Cas9 plasmid at 42°C. Fluorescence microscopy validated the successful generation of the Ty21a glms‐sfgfp reporter strain. For experimental applications, the Ty21a sfgfp strain was cultured in antibiotic‐free LB broth at 37°C with shaking until reaching mid‐log phase (OD600 = 0.9) and then maintained at 4°C for short‐term storage.

### 2.3. Experimental Oral Infection of *B. germanica* With *Salmonella* Typhi Ty21a sfgfp Strain

A total of 100 adult male *B. germanica* were individually housed in sterile disposable containers. Prior to experimentation, all subjects underwent a 3‐day starvation period with no access to food or water. Each cockroach was then orally inoculated with 10 μL of *Salmonella* Typhi Ty21a sfgfp suspension (OD_600_ = 0.9) using a micropipette to ensure successful inoculation. After inoculation, *B. germanica* were transferred to new sterile containers for individual rearing and provided with tap water and rodent diet. At designated time points, fecal samples and midgut tissues were collected for fluorescence microscopy to quantify the dynamics of Ty21a sfgfp within cockroaches.

### 2.4. Fecal–Oral Infection Model: Inoculation of BALB/c Mice With Cockroach‐Derived Ty21a sfgfp

**FIGURE 1:**
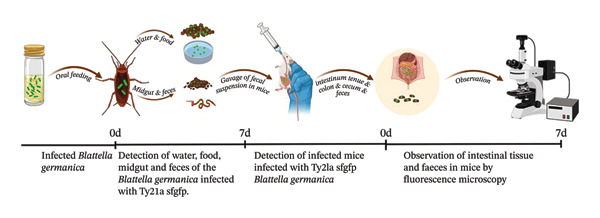
Experimental workflow. Adult male *B. germanica* (*n* = 100) was orally inoculated with fluorescently labeled *S.* typhi Ty21a sfgfp (10 μL, OD_600_ = 0.9). Midgut tissues and feces were collected at indicated time points for fluorescence microscopy and RFU quantification. For environmental contamination assays, water and food in confined chambers were sampled to assess pathogen deposition. For the murine fecal–oral exposure assay, feces with peak fluorescence (24 h) were homogenized in PBS and administered to BALB/c mice by gavage (100 μL). Mouse intestinal compartments and feces were collected over time (0.5 h–7 d) for fluorescence detection.

In recent studies, *Salmonella*‐infected *B. germanica* can facilitate horizontal pathogen transmission to conspecifics through contaminated water sources, food substrates, and fecal matter [23, 33]. Another research showed that cockroach feces serve as effective vehicles for disseminating potentially pathogenic bacterial strains [39]. Building upon this evidence, we hypothesized that Ty21a sfgfp–exposed *B. germanica* could contribute to mammalian exposure through a fecal–oral contamination pathway. To model ingestion of fecal‐contaminated material under enclosed conditions, fecal samples were collected from Ty21a sfgfp–exposed cockroaches at 24 h postinoculation (≈0.65 g) and homogenized in sterile phosphate‐buffered saline (PBS) to create a standardized fecal suspension. Viable Ty21a sfgfp in the suspension was confirmed and quantified by serial dilution and plating on LB agar (colony‐forming unit [CFU]), and fluorescence intensity (relative fluorescence unit [RFU]) was recorded by microscopy prior to gavage. BALB/c mice (*n* = 15 per group) were administered 100 μL of fecal suspension by oral gavage (exposed group), while negative‐control mice (NC; *n* = 15) received an equal volume of sterile PBS. Intestinal tissues (small intestine, cecum, and colon) and feces were collected at predetermined intervals (0.5, 3, 6, 12, 24 h, 4, and 7 d postexposure) for fluorescence microscopy and, when indicated, CFU enumeration (Figure 1). Each experiment was repeated three times to ensure reproducibility and reliability of results.

### 2.5. Viable Bacterial Quantification and Correlation With Fluorescence Intensity

To confirm that fluorescence signals reflected viable Ty21a sfgfp bacteria, selected samples (cockroach midgut homogenates, cockroach feces, mouse intestinal contents, and mouse feces) were serially diluted in PBS and plated on LB agar to determine CFUs. In parallel, fluorescence images were captured under identical acquisition settings and RFU were quantified in ImageJ. Linear regression between CFU and RFU was performed in GraphPad Prism.

### 2.6. Data Analysis and Statistics

Quantitative analysis of fluorescence images was performed using ImageJ (National Institutes of Health, NIH). Statistical analyses were conducted in GraphPad Prism 8.3 (GraphPad Software, USA). Data are presented as mean ± SEM from three independent experiments unless, otherwise, stated. For comparisons between two groups, a two‐tailed unpaired *t*‐test was used. For comparisons among multiple groups or time points, one‐way ANOVA with post hoc multiple comparisons was applied. The a priori significance threshold was set at *p* < 0.05.

## 3. Results

### 3.1. *B. germanica* Promotes the Environmental Transmission of Ty21a sfgfp to Water and Food

**FIGURE 2:**
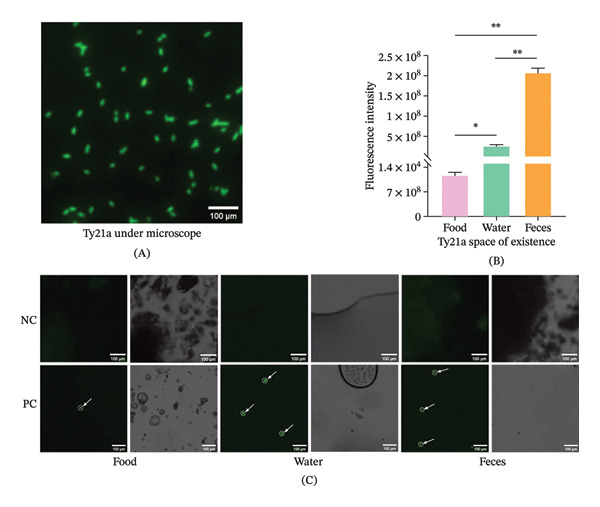
Environmental contamination mediated by Ty21a sfgfp–infected *B. germanica*. (A) Representative fluorescence micrograph of Ty21a sfgfp captured under identical acquisition settings. (B) Schematic of the confined chamber assay used to evaluate deposition of Ty21a sfgfp on water and food substrates. (C) Fluorescence (RFU) recovered from water, food, and cockroach feces at 1/4 and 1/2 day postinoculation (mean ± SEM, three independent experiments). Statistical analysis: one‐way ANOVA with multiple comparisons. ^∗^ *p* < 0.05; ^∗∗^ *p* < 0.01; ^∗∗∗^ *p* < 0.001; and ^∗∗∗∗^ *p* < 0.0001. NC, negative control.

**FIGURE 3:**
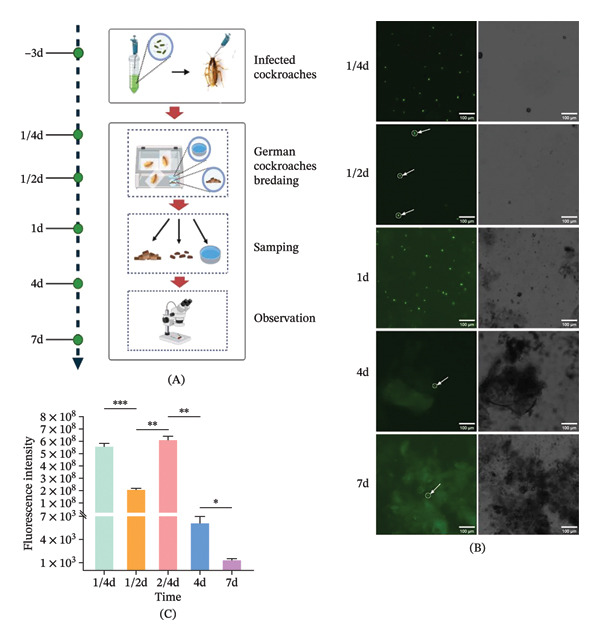
Fecal excretion dynamics of Ty21a sfgfp in *B. germanica*. (A) Workflow for fecal collection and fluorescence imaging. (B) Representative fluorescence images of fecal deposits collected at indicated time points postinoculation. (C) Quantification of fecal fluorescence intensity (RFU) over time (mean ± SEM, three independent experiments). Statistical analysis: one‐way ANOVA with multiple comparisons. ^∗^ *p* < 0.05; ^∗∗^ *p* < 0.01; ^∗∗∗^ *p* < 0.001; and ^∗∗∗∗^ *p* < 0.0001.

The genetically engineered Ty21a strain, expressing GFP, exhibited characteristic short, rod‐shaped morphology under fluorescence microscopy, with distinct green fluorescence emission (Figure 2A). In this experiment, individual *B. germanica* inoculated with Ty21a sfgfp were maintained in isolated chambers provided with sterile tap water and rat food. Environmental samples were collected at the 1/4 day and 1/2 day intervals postexposure for fluorescence microscopic analysis (Figure 3A). The results showed a significant difference (*p* = 0.0107, ANOVA) in Ty21a sfgfp load between water and food samples, with measured values of 2.63 × 10^7^ RFU and 1.18 × 10^4^ RFU at the 1/2 day, respectively (Figure 2B, C). Negative control (NC) groups showed no target fluorescence signal (at the same excitation/emission wavelengths as experimental groups) was detected in NCs throughout the experimental period (Figure 2C). These findings showed that the capacity of Ty21a sfgfp–infected *B. germanica* to contaminate environmental resources, establishing their potential role in the transmission of pathogens.

### 3.2. Dynamics of Ty21a sfgfp in the *B. germanica* Midgut

**FIGURE 4:**
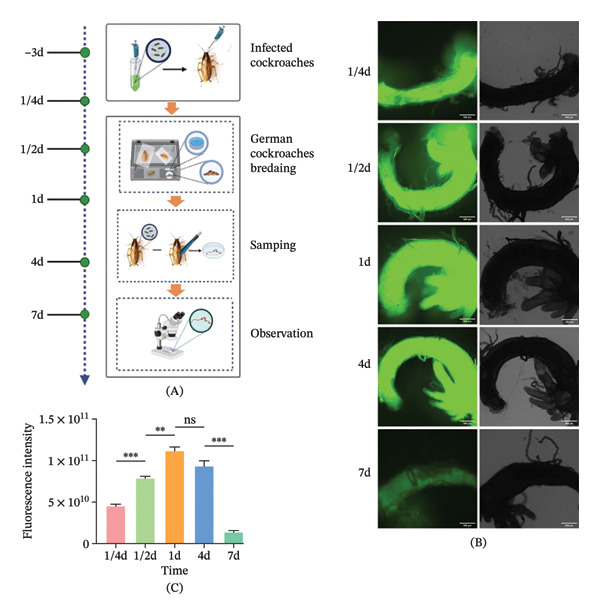
Midgut bacterial signal detection dynamics of Ty21a sfgfp in *B. germanica*. (A) Experimental schema for midgut dissection and fluorescence imaging. (B) Representative midgut fluorescence images collected at indicated time points postinoculation. (C) Quantification of midgut fluorescence intensity (RFU) over time (mean ± SEM, three independent experiments). Statistical analysis: one‐way ANOVA with multiple comparisons. ^∗^ *p* < 0.05; ^∗∗^ *p* < 0.01; ^∗∗∗^ *p* < 0.001; and ^∗∗∗∗^ *p* < 0.0001.

To study the dynamics of Ty21a sfgfp in *B. germanica* midgut, midgut tissues were aseptically dissected for fluorescence microscopic observation of Ty21a sfgfp dynamics (Figure 4A). The results revealed a peak between 1/4‐day and 1‐day postinfection (dpi), with fluorescence intensities ranging from 4.52 × 10^10^ to 1.11 × 10^11^ RFUs. Further analysis revealed a statistically significant increasing trend in bacterial load during the 1/2‐1 day period (*p* = 0.0091, ANOVA). The bacterial load exhibited a gradual decline thereafter, decreasing to 9.35 × 10^10^ RFU by 4dpi (*p* = 0.0898, ANOVA) and reaching baseline levels (1.37 × 10^10^ RFU) at 7 dpi (*p* = 0.0084, ANOVA) (Figure 4B, C). NC groups showed no target fluorescence signal was detected in NCs throughout the experimental period (Figure 4B).

These findings established the presence of Ty21a sfgfp in the *B. germanica* midgut. This bacterial signal detection phenomenon provides critical insights into host–pathogen interactions at the insect–microbe interface.

### 3.3. Monitoring of Fecal Ty21a sfgfp Excretion in *B. germanica*

To characterize the fecal excretion dynamics of Ty21a sfgfp in *B. germanica*, the study conducted systematic fecal sample collection and fluorescence microscopic analysis (Figure 3A). This study revealed that initial detection of Ty21a sfgfp at 1/4 day postinfection (dpi), with a fluorescence intensity of 5.61 × 10^8^ RFU. The bacterial load exhibited dynamic fluctuations, decreasing to 2.08 × 10^8^ RFU at 1/4–1/2 dpi (*p* = 0.0008, ANOVA), followed by a secondary peak of 6.12 × 10^8^ RFU at 1/2 ‐1 dpi (*p* = 0.0010, ANOVA). Subsequently, the fluorescence intensity was 6.13 × 10^3^ RFU at 1‐4 dpi (*p* = 0.0029, ANOVA). Persistent bacterial signal was evidenced by detectable fluorescence (1.33× 103 RFU) at 4–7 dpi (*p* = 0.0394, ANOVA) (Figure 3B, C). Control groups fed with sterile LB broth showed that no target fluorescence signal (at the same excitation/emission wavelengths as experimental groups) was detected in NCs throughout the experimental period (Figure 3B). These results showed that Ty21a sfgfp maintains persistent in *B. germanica*, for a minimum of 7 dpi postinfection.

### 3.4. Ty21a sfgfp Dissemination From *B. germanica* to BALB/c Mice

**FIGURE 5:**
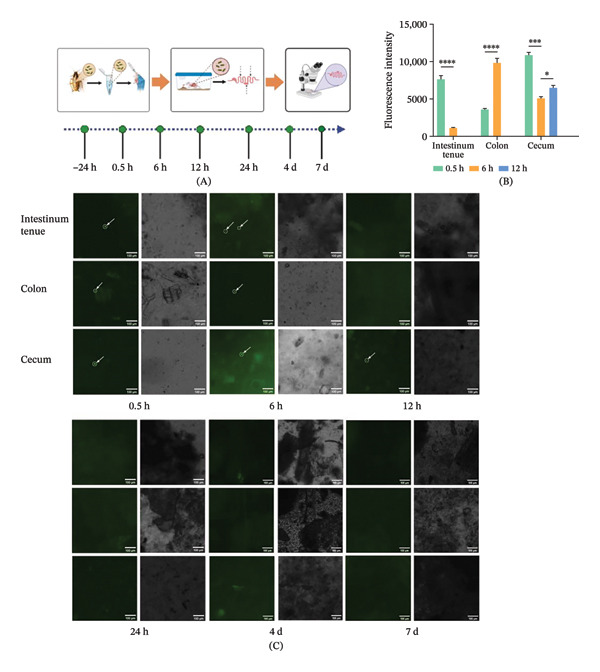
Detection of Ty21a sfgfp in the intestinal tract of BALB/c mice following ingestion of cockroach‐derived fecal suspension. (A) Schematic of sampling sites (small intestine, cecum, and colon). (B) Fluorescence intensity (RFU) quantified in each intestinal compartment at 0.5, 3, 6, 12, 24 h, 4, and 7 d postgavage. (C) Clearance kinetics of Ty21a sfgfp from the murine gut. Data are shown as mean ± SEM (*n* = 15 mice per group; three independent experiments). Statistical analysis: *t*‐test for small intestine and colon, and one‐way ANOVA for cecum with multiple comparisons. ^∗^ *p* < 0.05; ^∗∗^ *p* < 0.01; ^∗∗∗^ *p* < 0.001; and ^∗∗∗∗^ *p* < 0.0001. N indicates no detectable fluorescence signal.

To further assess whether Ty21a sfgfp shed by infected *B. germanica* could reach a mammalian gastrointestinal tract under a fecal–oral exposure scenario, we administered a cockroach‐derived fecal suspension to BALB/c mice (Figure 5A). Feces collected at 24 h postinoculation (6.12 × 10^8^ RFU) were homogenized in sterile PBS to generate a standardized suspension (3.79 × 10^7^ RFU) and gavaged to mice. Fluorescence signals were detected in intestinal compartments shortly after gavage, with cecal values ranging from 1.13 × 10^3^ to 1.08 × 10^4^ RFU and persisting up to 12 h. No fluorescence was detectable at 24 h and remained undetectable at later monitoring time points (4 d and 7 d) (Figure 5B, C).

**FIGURE 6:**
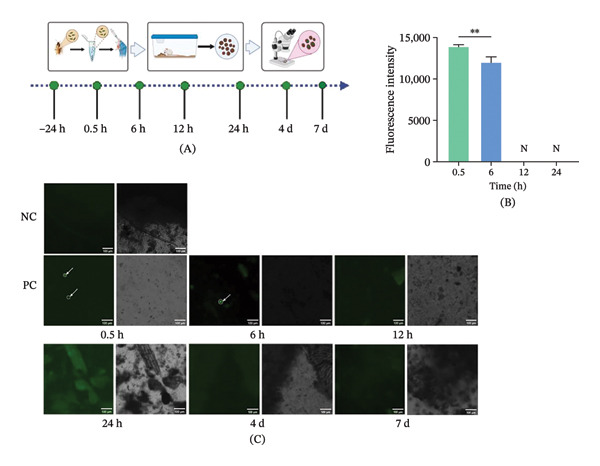
Fecal detection of Ty21a sfgfp in BALB/c mice after gavage with cockroach‐derived fecal suspension. (A) Experimental scheme and sampling time points. (B) Fluorescence intensity (RFU) detected in feces at 0.5, 3, 6, 12, 24 h, 4, and 7 d postgavage. (C) Time‐dependent fecal shedding profile of Ty21a sfgfp. Data are shown as mean ± SEM (*n* = 15 mice per group; three independent experiments). Statistical analysis: two‐tailed *t*‐test for exposed vs. NC comparisons. ^∗^ *p* < 0.05; ^∗∗^ *p* < 0.01; ^∗∗∗^ *p* < 0.001; and ^∗∗∗∗^ *p* < 0.0001. N indicates no detectable fluorescence signal.

To further characterize the intestinal clearance and shedding dynamics of Ty21a sfgfp in murine hosts, fecal samples were collected after oral exposure (Figure 6A). Fluorescence was detectable in mouse feces at 0.5–6 h postgavage, with intensities of 1.20 × 10^4^ to 1.39 × 10^4^ RFU (*p* = 0.0094, *t-*test). No target fluorescence signal was detected from 12 h onward, remaining negative through 24 h, 4, and 7 d postinoculation (Figure 6B, C).

### 3.5. Correlation Between Fluorescence Intensity (RFU) and CFU

**FIGURE 7:**
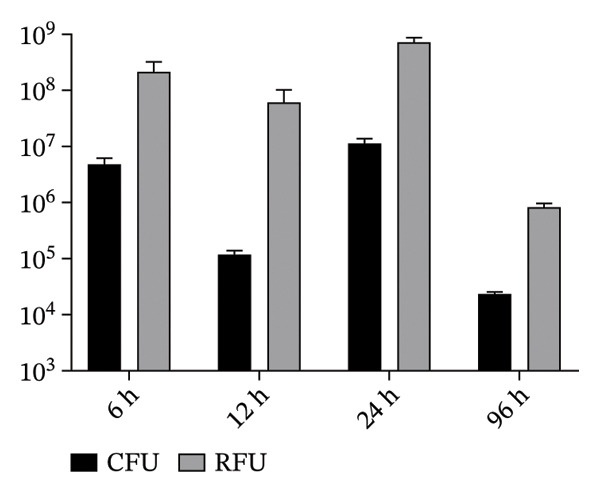
Correlation between fluorescence intensity (RFU) and viable Ty21a sfgfp counts (CFU). Representative samples were quantified by fluorescence microscopy (RFU) and by serial dilution plating (CFU). Scatter plot shows linear regression with the fitted equation, *R* ^2^, and *p* value (three independent experiments).

To validate that fluorescence‐based quantification reflected viable Ty21a sfgfp, CFU enumeration was performed in parallel with RFU measurement for representative cockroach‐ and mouse‐derived samples. Linear regression analysis showed a significant positive correlation between CFU and RFU (*R* ^2^ = 0.78, *p* < 0.05), described by the equation CFU = 0.01424 × RFU + 612,887 (Figure 7). These results support the use of fluorescence intensity as a proxy for viable bacterial abundance in this study.

## 4. Discussion

In this study, Ty21a sfgfp–exposed *B. germanica* contaminated water and food resources within confined chambers, supporting the view that synanthropic cockroaches can act as mechanical vectors that disseminate enteric bacteria in enclosed environments. *B. germanica* frequently forages in food preparation and healthcare areas and can move between contaminated substrates (sewage, waste, and decaying organic material) and human‐associated surfaces, providing opportunities for indirect food‐ and waterborne exposure. Recent field surveys and systematic assessments continue to document cockroach infestations and pathogen carriage in high‐risk settings, reinforcing the importance of integrated pest management and hygiene as complementary public health measures [25–28].

Our time‐course analyses showed that Ty21a sfgfp persisted in the cockroach midgut and feces for up to 7 days, consistent with prior observations that ingested *Salmonella* can survive and be shed for multiple days in cockroaches. Mechanistically, the cockroach gut can provide a permissive niche in which pathogen survival is shaped by host strain, infectious dose, and interactions with the gut microbiota and innate immune responses [22, 40]. Prolonged shedding increases the likelihood of contaminating communal food and water sources and, consequently, the probability of fecal–oral exposure in crowded or poorly sanitized settings.

In the murine exposure assay, ingestion of cockroach‐derived fecal suspension led to rapid but transient detection of Ty21a sfgfp signals in the intestinal tract and feces, with fluorescence detectable up to 12 h and absent thereafter (up to 7 d). Notably, this gavage model was used to mimic ingestion of cockroach‐feces–contaminated material and represents a controlled, high‐dose exposure scenario rather than an observation of natural transmission; in preliminary cohousing experiments (mice housed with infected cockroaches), we did not detect Ty21a sfgfp in mice. The transient signals observed here are also consistent with use of the attenuated Ty21a vaccine strain and host clearance mechanisms. To strengthen the interpretability of fluorescence measurements, we confirmed a positive correlation between RFU and viable bacterial counts (CFU) (Figure 7), indicating that the recorded signals reflected viable bacteria rather than fluorescence alone. Together, these findings support a plausible fecal–oral exposure pathway mediated by *B. germanica*, while underscoring the need for future studies using wild‐type clinical isolates and more naturalistic exposure designs to quantify transmission efficiency and disease risk.

Future work should evaluate a broader range of *Salmonella* serovars and doses, incorporate environmental variables (temperature, humidity, and sanitation), and apply complementary readouts (culture, molecular detection, and genomic typing) to strengthen source attribution and risk assessment in real‐world settings [35–38].

## 5. Conclusions

This study demonstrates that Ty21a sfgfp acquired from the environment can persist in the gut of *B. germanica* for up to 7 days and be excreted in feces, contaminating nearby food and water resources. Under an experimental fecal–oral exposure model, the ingestion of cockroach‐derived fecal suspension resulted in transient detection of Ty21a sfgfp in the murine gastrointestinal tract (≤ 12 h) with no detectable signal thereafter (up to 7 days). These findings support the potential of synanthropic cockroaches to facilitate fecal–oral exposure of mammals to *Salmonella* in enclosed environments and underscore the importance of sanitation and cockroach control to reduce contamination risks.

## Funding

This research did not receive any specific grant from funding agencies in the public, commercial, or not‐for‐profit sectors.

## Disclosure

No funding source had any role in study design; collection, analysis, or interpretation of data; writing of the report; or the decision to submit the manuscript for publication.

## Ethics Statement

All animal procedures were approved by the guidance of the Institutional Animal Care and Use Committee (IACUC) of the State Key Laboratory of Pathogen and Biosecurity (IACUC‐DWZX‐2024‐P302) and were conducted in accordance with national and institutional guidelines for the care and use of laboratory animals.

## Conflicts of Interest

The authors declare no conflicts of interest.

## Data Availability

The data that support the findings of this study are available from the corresponding author upon reasonable request.
